# Metadata Conditioning via Feature-Wise Linear Modulation for Multi-Domain 3D Abdominal Segmentation: A Comparative Study Between U-Net and nnU-Net

**DOI:** 10.3390/s26134109

**Published:** 2026-06-29

**Authors:** Juan F. Garrido-Martínez, Javier M. Garrido-López, Juan F. Zapata-Pérez, Juan Martínez-Alajarín

**Affiliations:** Escuela Técnica Superior de Ingeniería Industrial, Campus Muralla del Mar, Universidad Politécnica de Cartagena, Member of European University of Technology EUT+, C/Doctor Fleming, s/n, 30202 Cartagena, Spain; javier.garrido@upct.es (J.M.G.-L.); juan.zapata@upct.es (J.F.Z.-P.); juanc.martinez@upct.es (J.M.-A.)

**Keywords:** 3D abdominal segmentation, magnetic resonance imaging, feature-wise linear modulation, nnU-Net, domain shift, metadata conditioning

## Abstract

Automatic 3D abdominal organ segmentation in magnetic resonance imaging presents a major challenge: data heterogeneity, caused by differences in acquisition protocol, contrast, spatial resolution, and imaging characteristics, which can degrade model generalization. This work investigates the use of FiLM (Feature-wise Linear Modulation) as a metadata-conditioning mechanism to improve the contextual adaptation of 3D segmentation networks. To this end, two reference architectures, U-Net and nnU-Net v2, were compared in both their baseline versions and their FiLM-conditioned variants. The evaluation was carried out on the CHAOS and AMOS datasets, as well as on a combined CHAOS+AMOS setting to analyze multi-domain robustness. The results show that the effect of FiLM clearly depends on the underlying architecture. In U-Net, FiLM produced favourable quantitative trends in the final post-processed predictions, with a small gain in CHAOS, a clearer improvement in AMOS, and the largest effect in the combined scenario, where the increase reached +0.0215 in clean Dice, together with reductions of −6.66 mm in HD95 and −3.85 mm in ASD. However, these improvements were not uniform across all metrics and organs. In contrast, in nnU-Net the conditioning did not provide consistent benefits and reduced performance in some scenarios, particularly in AMOS. In addition, when post-processing was applied to remove islands and disconnected components, its impact was much greater in U-Net than in nnU-Net, indicating that the latter produced cleaner and more stable segmentations directly from the raw output. Overall, this work suggests that FiLM can be useful to reinforce more generic architectures such as U-Net, especially when problem heterogeneity increases, but it was not consistently beneficial for nnU-Net, whose baseline already constituted the most robust and best-balanced model in the study. These results support the idea that the value of metadata conditioning is not universal, but instead depends on the base architecture and the generalization scenario under consideration.

## 1. Introduction

Automatic segmentation of abdominal organs in three-dimensional magnetic resonance imaging (MRI) is an important task in medical image analysis due to its relevance in clinical practice and biomedical research. Accurate delineation of structures such as the liver, spleen, and kidneys can support surgical and radiotherapy planning, anatomical quantification, disease monitoring, and patient-specific assessment. However, manual segmentation of 3D abdominal images remains time-consuming and observer-dependent, particularly when volumetric studies require slice-by-slice annotation. These limitations have motivated the development of automatic segmentation methods capable of producing accurate, reproducible, and efficient organ masks [1,2].

Abdominal MRI remains a challenging segmentation scenario because organs vary considerably in shape, size, position, and relationship with surrounding tissues. In addition, organ boundaries may be difficult to distinguish due to low soft-tissue contrast, partial volume effects, respiratory motion, intensity inhomogeneity, and the similar appearance of adjacent structures [3]. These difficulties are further amplified by acquisition-related variability. Unlike computed tomography, MRI does not provide a universal absolute intensity scale, and image appearance may change substantially depending on sequence type, scanner configuration, spatial resolution, reconstruction process, contrast characteristics, and protocol-specific parameters [4].

This variability can produce a domain shift, understood as a mismatch between the data distribution used during training and the distribution encountered during validation, testing, or real-world deployment. In medical imaging, such shifts may arise from differences in scanner manufacturer, acquisition sequence, spatial resolution, reconstruction settings, or contrast properties. Consequently, a segmentation model trained under specific conditions may experience reduced performance when applied to images acquired under different conditions. This problem is especially relevant in multi-domain scenarios, where images from different datasets or acquisition protocols are combined within the same segmentation task [5,6,7,8].

Deep learning has substantially improved medical image segmentation, particularly through convolutional encoder–decoder architectures [1,9]. Among them, U-Net and its three-dimensional extensions have become widely used baselines for volumetric biomedical segmentation because they combine contextual feature extraction with spatial resolution recovery [10,11]. However, standard U-Net architectures do not explicitly account for acquisition-dependent variability, since all input images are processed using the same learned operations regardless of sequence type, dataset domain, or intensity characteristics. In contrast, nnU-Net has emerged as a strong reference framework for biomedical image segmentation by automatically adapting key elements of the segmentation pipeline to each dataset [12]. For this reason, recent work has emphasized that new architectural proposals should be compared against strong baselines such as nnU-Net to avoid overestimating methodological improvements [13].

One possible strategy to improve robustness under heterogeneous acquisition conditions is to provide the model with auxiliary information describing the acquisition context or global appearance of each image. Instead of forcing the network to infer all sources of variability from the image alone, metadata can be used to condition its internal representations. Feature-wise Linear Modulation (FiLM) provides a simple and flexible mechanism for this purpose by using an external conditioning vector to generate feature-wise scaling and shifting parameters that modulate intermediate feature maps [14]. Although FiLM was originally introduced in visual reasoning tasks, its general formulation makes it suitable for medical imaging scenarios where acquisition-related or image-derived metadata may help adapt learned representations [15].

In abdominal MRI segmentation, FiLM is particularly appealing because differences in acquisition sequence, contrast, and intensity distribution can affect the interpretation of anatomical structures. However, the benefit of metadata conditioning should not be assumed to be universal. Its effectiveness may depend on the selected metadata, the degree of heterogeneity, the location of the modulation layers, and the robustness of the baseline architecture. In a standard 3D U-Net, FiLM may provide an additional adaptation mechanism, whereas in nnU-Net part of the variability may already be handled by its automatic preprocessing, planning, normalization, and training configuration.

To investigate this question, this work evaluates metadata conditioning through FiLM for 3D abdominal organ segmentation in MRI. Two reference architectures are compared: a standard 3D U-Net and nnU-Net v2. Each architecture is evaluated in its baseline form and in a FiLM-conditioned variant. The experiments are performed on two public abdominal MRI benchmarks, CHAOS and AMOS, as well as on a combined CHAOS+AMOS scenario designed to increase acquisition-related and dataset-related heterogeneity [16,17,18]. In addition, both raw and connected-component post-processed predictions are analyzed in order to distinguish the intrinsic stability of the model outputs from the final performance of the complete segmentation pipeline.

The main contributions of this work are: first, the integration and evaluation of FiLM as a metadata-conditioning mechanism for 3D abdominal MRI segmentation; second, the systematic comparison of standard 3D U-Net and nnU-Net v2, with and without FiLM, across CHAOS, AMOS, and CHAOS+AMOS; third, the analysis of metadata conditioning under different levels of acquisition-related heterogeneity; and fourth, the evaluation of connected-component post-processing by comparing raw and clean predictions. Rather than assuming that metadata conditioning provides a universal improvement, this study analyzes when FiLM is beneficial and when its contribution is limited by the strength of the underlying segmentation framework.

The remainder of this paper is organized as follows. Section 2 reviews related work on medical image segmentation, nnU-Net, metadata conditioning, FiLM, and domain shift in medical imaging. Section 3 describes the methodology, including datasets, preprocessing, metadata extraction, model training, post-processing, and evaluation. Section 4 presents the experimental results, Section 5 discusses the main findings, and Section 6 summarizes the conclusions and future research directions.

## 2. Related Work

Deep learning has become the dominant approach for medical image segmentation, particularly after the introduction of fully convolutional networks for dense prediction tasks [19]. In biomedical imaging, U-Net became one of the most widely used encoder–decoder architectures due to its skip connections and its ability to combine contextual information with spatial detail [10]. This design was later extended to volumetric data through 3D U-Net, allowing the model to exploit inter-slice anatomical continuity [11], while other architectures such as V-Net further consolidated fully convolutional 3D segmentation and Dice-based optimization for imbalanced medical data [20]. Subsequent methods have introduced dense skip pathways, attention gates, transformer-based global context modeling, and self-supervised pretraining, as illustrated by UNet++, Attention U-Net, TransUNet, Swin UNETR, and Models Genesis [21,22,23,24,25]. Despite this diversity, U-Net-like architectures remain strong baselines for medical and abdominal organ segmentation [1,26].

Among current reference methods, nnU-Net has become one of the most influential frameworks in biomedical image segmentation. Rather than proposing only a fixed architecture, nnU-Net automatically adapts key elements of the segmentation pipeline, including preprocessing, resampling, normalization, network configuration, training, inference, and post-processing, to the properties of each dataset [12]. Its importance as a benchmark has also been emphasized in recent work on rigorous validation of 3D medical segmentation methods [13]. This is particularly relevant for the present study, since the effect of metadata conditioning should be evaluated not only against a standard 3D U-Net, but also against a strong and carefully optimized framework such as nnU-Net v2.

Abdominal MRI segmentation remains challenging because image appearance is strongly affected by acquisition sequence, scanner configuration, contrast properties, spatial resolution, and intensity distribution. Recent works have addressed this problem from complementary perspectives. Zhuang et al. proposed MRISegmenter for automated multiorgan and structure segmentation in abdominal MRI, reflecting the growing interest in robust MRI-specific abdominal segmentation systems [27]. Ciausu et al. investigated the use of synthesized data generated from CT labels to support abdominal MRI organ segmentation, addressing the limited availability of voxel-level MR annotations and the difficulty of learning from heterogeneous MRI data [28]. Public benchmarks such as CHAOS and AMOS provide useful data sources for studying these challenges, since they include abdominal MRI data acquired under different sequences, protocols, and clinical conditions [16,17].

Most segmentation models, however, still process the medical image as the only input and do not explicitly use contextual information associated with the acquisition process. Metadata conditioning aims to incorporate auxiliary information, such as acquisition-related or image-derived descriptors, so that the network can adapt its internal representations to each case. Related ideas have been explored through conditional normalization and feature modulation mechanisms [29,30]. Feature-wise Linear Modulation (FiLM) provides a simple implementation of this idea by using an external conditioning vector to generate feature-wise scaling and shifting parameters for intermediate feature maps [14]. Its simplicity and low computational cost make it attractive for medical imaging, where it is often desirable to preserve a validated segmentation backbone while adding lightweight contextual adaptation [31]. In medical segmentation, Lemay et al. showed that metadata can be integrated through low-cost feature modulation [15], while Khan et al. proposed a metadata-aware cardiac segmentation framework to improve robustness under acquisition- and patient-related variability [32]. These studies suggest that metadata can be useful, although its benefit may depend on the metadata type, anatomy, architecture, modulation strategy, and degree of heterogeneity.

The motivation for metadata conditioning is closely related to domain shift in medical imaging. Recent surveys on domain adaptation and domain generalization have highlighted that robustness to out-of-distribution data and inter-site variability remains a major challenge for medical image analysis systems [5,6]. In segmentation, this mismatch may cause performance degradation when models are applied to images acquired with different scanners, protocols, or population characteristics. Karani et al. showed that CNN-based segmentation models can degrade under such shifts and proposed test-time adaptation as a strategy to improve robustness [7]. Multi-domain and multi-dataset learning can also improve generalization by exposing models to different sources, although it introduces challenges related to resolution, intensity distribution, annotation protocol, and label availability [18]. In this context, FiLM can be interpreted as a lightweight strategy for adapting internal representations according to acquisition-related or image-derived descriptors, without replacing dedicated domain adaptation or domain generalization methods.

Recent segmentation research has also explored alternative strategies to improve representation robustness, including knowledge distillation, contrastive learning, and prototype-based learning. Zhou et al. proposed MRKD-PBCL, a class-incremental semantic segmentation method based on multi-level region-wise knowledge distillation and prototype balanced contrastive learning [33]. This work is relevant because it reflects the increasing interest in preserving useful representations and improving class separation under changing learning conditions. However, it addresses a different problem from the present study: MRKD-PBCL focuses on learning new classes while retaining previous knowledge, whereas our work focuses on 3D abdominal MRI segmentation under acquisition-related and dataset-related variability. Therefore, MRKD-PBCL is not considered a direct baseline here, but it provides useful context for positioning FiLM-based metadata conditioning among recent strategies aimed at improving segmentation robustness.

Evaluation methodology is also important because no single metric fully captures segmentation quality. The Dice Similarity Coefficient is widely used to quantify volumetric overlap [34], but it is commonly complemented with surface-distance metrics such as Hausdorff Distance and Average Surface Distance to assess boundary accuracy and spatial agreement [35,36]. In addition, connected-component post-processing is frequently used to remove small disconnected regions or isolated false positives, and previous work has shown that automatic post-processing can improve abdominal MRI segmentation by correcting common prediction errors [37]. Evaluating both raw and post-processed predictions can therefore help distinguish the intrinsic stability of the model output from the final performance of the complete segmentation pipeline.

Unlike previous FiLM-based medical segmentation studies, which mainly showed that metadata can be incorporated through feature modulation [15,32], this work evaluates FiLM as an architecture-dependent strategy. Specifically, we compare its effect in a generic 3D U-Net and in nnU-Net v2, whose self-configuring pipeline already adapts preprocessing, normalization, planning, training, and inference [12]. Thus, the main conceptual contribution is to assess when metadata conditioning remains useful across different levels of built-in adaptability and across single- and multi-domain abdominal MRI settings.

Overall, previous work shows that U-Net-like architectures remain strong foundations for biomedical segmentation, nnU-Net represents a robust reference framework, abdominal MRI segmentation is still affected by acquisition variability and limited annotation availability, and FiLM provides a lightweight mechanism for incorporating contextual information into neural networks. At the same time, recent methods such as MRKD-PBCL show that robust segmentation increasingly relies on improved representation learning, although through strategies different from metadata conditioning. This motivates the present study, which evaluates whether FiLM-based metadata conditioning provides measurable benefits in 3D abdominal MRI segmentation, and whether these benefits differ between a standard 3D U-Net and a strong nnU-Net v2 baseline across CHAOS, AMOS, and a combined CHAOS+AMOS scenario.

## 3. Materials and Methods

This section describes the experimental protocol used to evaluate FiLM-based metadata conditioning for 3D abdominal MRI segmentation. Four model configurations were compared: a baseline 3D U-Net, a FiLM-conditioned 3D U-Net, a baseline nnU-Net v2, and a FiLM-conditioned nnU-Net v2. Experiments were conducted in three scenarios: CHAOS, AMOS, and a combined CHAOS+AMOS setting. The individual datasets were used to assess model behavior under dataset-specific conditions, whereas the combined scenario was designed to evaluate robustness under increased multi-domain heterogeneity.

This design allowed us to analyze whether FiLM provides additional benefit depending on the underlying architecture and data variability. The standard 3D U-Net was used as a generic encoder–decoder baseline, while nnU-Net v2 was included as a strong self-configuring framework. In all scenarios, both raw and connected-component post-processed predictions were evaluated to assess segmentation accuracy and structural stability.

### 3.1. Datasets and Acquisition Context

Two publicly available abdominal imaging datasets were used: CHAOS and AMOS [16,17]. Both datasets are commonly used for the development and evaluation of automatic abdominal organ segmentation methods. In this study, only magnetic resonance imaging data were considered. From a biomedical sensing perspective, each MRI volume can be interpreted as the output of an acquisition system in which the measured signal is shaped by scanner hardware, pulse sequence, spatial sampling, reconstruction, and protocol-specific parameters. Therefore, the input data used in this study are not only image volumes, but sensor-derived biomedical measurements whose appearance may vary across acquisition settings.

The CHAOS MRI subset included 60 volumetric series from 20 subjects, with three acquisitions per subject: T1 in-phase, T1 out-of-phase, and T2-SPIR. These acquisitions introduce differences in contrast and tissue appearance within a relatively controlled dataset structure. The AMOS MRI subset included 60 MRI cases with available annotations for the target organs. Compared with CHAOS, AMOS represents a more heterogeneous setting, with images acquired under more diverse clinical and acquisition conditions. However, detailed acquisition metadata were not available in a homogeneous way across all cases and both datasets, which limited the possibility of explicitly analyzing scanner- or protocol-specific effects. In addition to the individual CHAOS and AMOS experiments, a combined CHAOS+AMOS scenario was defined by integrating MRI cases from both datasets, allowing the evaluation of a single model across two public abdominal MRI sources.

A common five-class label space was used across all experiments: background (0), liver (1), right kidney (2), left kidney (3), and spleen (4). Since the original annotation conventions differed between datasets, all masks were remapped before training and evaluation to ensure a consistent segmentation task. In CHAOS, the source labels were 63 for liver, 126 for right kidney, 189 for left kidney, and 252 for spleen. In AMOS, the corresponding source labels were 6, 2, 3, and 1, respectively.

A fixed 70/15/15 train/validation/test split was used in all experiments to ensure a direct comparison between baseline and FiLM-conditioned models under identical data partitions. In CHAOS, the split was performed at subject level to avoid data leakage between the different MRI sequences from the same subject. In AMOS, each case was treated as an independent volumetric study. In the combined CHAOS+AMOS scenario, the test set was balanced between domains, including 9 CHAOS and 9 AMOS cases. The dataset composition and split are summarized in Table 1.

All data used in this study were obtained from publicly available and anonymized benchmark datasets. No private, proprietary, or internally collected clinical data were used, and the study did not involve direct patient recruitment, prospective data acquisition, or access to identifiable clinical information. Although this improves reproducibility, the experiments depend on the specific MRI subsets, label harmonization strategy, preprocessing pipeline, and fixed data partitions defined in this study.

### 3.2. Preprocessing, Metadata Extraction and Normalization

The preprocessing pipeline converted the original CHAOS and AMOS data into a common volumetric format suitable for training and evaluation. Since both datasets used different image formats and annotation conventions, dataset-specific preprocessing was first applied. The final output consisted of paired NIfTI volumes, including one MRI image and its corresponding multi-class segmentation mask.

For CHAOS, MR images were reconstructed from DICOM series while preserving the available spatial metadata, including voxel spacing, orientation, and origin. The original two-dimensional PNG masks were sorted, stacked into 3D label volumes, and remapped to the shared label space defined in Section 3.1. When the number of image and mask slices did not match, both volumes were center-cropped along the slice direction to obtain a consistent image-label pair. For AMOS, preprocessing started from the original NIfTI images and labels. Only MRI cases with valid ground-truth annotations were retained, and labels not included in the target organ set were assigned to background.

After dataset-specific preprocessing, all valid cases were registered in a unified metadata index containing the dataset origin, case identifier, image and label paths, sequence information when available, voxel spacing, and split assignment. Quality-control checks verified file existence, valid label values, shape consistency, affine compatibility, and non-empty foreground masks. This ensured that all models were trained and evaluated using consistent image-label pairs while preserving the acquisition-related variability required for the robustness analysis.

Metadata were extracted at case level to provide the FiLM-conditioned models with contextual information related to the acquisition setting or the global appearance of each MRI volume. The objective was not to introduce clinical variables, but to define image-level descriptors that could help the network adapt its internal representations to differences in sequence type, intensity distribution, contrast, and dataset domain.

Different metadata configurations were used depending on the experimental scenario, as summarized in Table 2. In CHAOS, sequence metadata were used because the acquisition sequence was explicitly available and represented a relevant source of appearance variability. The sequence was encoded using a three-dimensional one-hot vector indicating whether the input corresponded to T1 in-phase, T1 out-of-phase, or T2-SPIR. In AMOS and CHAOS+AMOS, intensity-based descriptors were selected to summarize global image characteristics in a dataset-independent way.

For AMOS, no categorical sequence information equivalent to the CHAOS MRI sequence labels was available. Therefore, image-derived intensity descriptors were used as metadata. These descriptors were selected because they can be computed directly from the input MRI volume and provide a compact summary of its global appearance. Specifically, the mean intensity, standard deviation, and robust lower and upper percentiles were used to characterize the overall signal level, intensity dispersion, and dynamic range of each case. The resulting variables were standardized and denoted as ${\mathrm{mean}}_{z}$, ${\mathrm{std}}_{z}$, $p05_{z}$, and $p95_{z}$.

For the combined CHAOS+AMOS scenario, foreground-based intensity descriptors were used instead of whole-volume statistics to better account for differences in background, field of view, cropping, and intensity distribution between datasets. The foreground region was estimated directly from each MRI volume by selecting finite non-background voxels, without using the ground-truth masks, thereby avoiding anatomical label leakage and allowing the same procedure to be applied during inference. Foreground intensities were robustly clipped using the 1st and 99th percentiles and rescaled to [0, 1]. The selected descriptors were the 25th and 75th percentiles, foreground mean, skewness, and a contrast ratio, summarizing the central intensity range, average signal level, distribution asymmetry, and relative contrast of the foreground region. The contrast ratio was defined as:

(1) $$\mathrm{contrast}\text{\_}\mathrm{ratio}\;=\;\frac{p_{75}\;-\;p_{25}}{p_{95}-p_{05}\;+\;\varepsilon }$$

where ε is a small numerical stability constant. A zero or near-zero value of $p_{95}\;-\;p_{05}$ is not expected in normal cases, but it could occur in degenerate or nearly constant foreground regions. Therefore, ε was retained to avoid numerical instability without affecting ordinary cases.

All continuous metadata variables were standardized before being provided to the FiLM modules. For each experimental scenario, the mean and standard deviation of each metadata variable were computed using only the training cases and then applied to the validation and test cases. This avoided information leakage from the evaluation data. For each raw continuous metadata variable *m*, its standardized version $m_{z}$ was computed as:

(2) $$m_{z}\;=\;\frac{m\;-\;\mu_{\mathrm{train}}}{\sigma_{\mathrm{train}}\;+\;\varepsilon }$$

where $\mu_{\mathrm{train}}$ and $\sigma_{\mathrm{train}}$ are the mean and standard deviation estimated from the training set, and ε is included for numerical stability. Categorical sequence metadata were not standardized because they were represented using one-hot encoding.

### 3.3. Baseline Architectures

Baseline models were included to establish reference segmentation performance without metadata conditioning. Two architectures were evaluated across CHAOS, AMOS, and CHAOS+AMOS: a standard 3D U-Net and nnU-Net v2. In both cases, only the preprocessed MRI volume was used as input, and the resulting performance served as the reference for comparison with the FiLM-conditioned variants.

#### 3.3.1. U-Net Baseline

The first baseline was a standard 3D U-Net implemented in PyTorch 2.8.0 for multi-class abdominal organ segmentation. The network received a single-channel MRI volume as input and produced voxel-wise logits for the five-class segmentation task. It followed a classical encoder–decoder structure with three encoder levels, a bottleneck, and a symmetric decoder connected through skip connections. In the encoder, spatial resolution was progressively reduced while feature representations evolved from low-level local patterns to more abstract contextual and anatomical features. The bottleneck provided the deepest contextual representation of the input volume. In the decoder, spatial resolution was progressively restored using transposed convolutions, while skip connections fused encoder and decoder features to support feature recovery at coarse, intermediate, and fine levels of detail.

**Figure 1:**
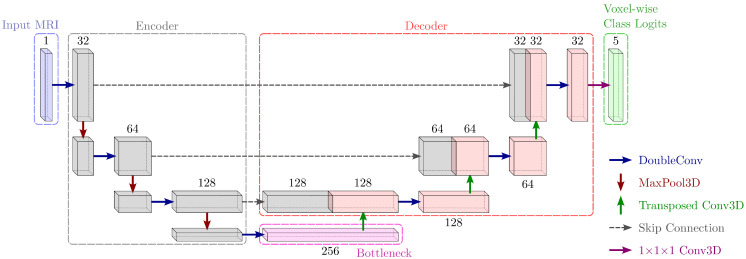
Diagram of the 3D U-Net baseline architecture, illustrating its classical encoder–decoder structure with three levels of downsampling and upsampling, and the integration of skip connections to fuse features from corresponding encoder and decoder stages. This design allows for the recovery of fine spatial details while capturing high-level contextual information. Numbers indicate the number of feature channels at each stage, and the color-coded arrows correspond to the operations shown in the legend.

Each convolutional block was implemented as a DoubleConv module composed of two consecutive Conv3D–InstanceNorm–LeakyReLU operations, using a kernel size of 3 and padding of 1. The base number of feature channels was set to 32 and doubled after each downsampling operation, reaching 256 channels at the bottleneck. A final 1 × 1 × 1 convolution mapped the decoder output to the five target classes. When required, center-cropping was applied before concatenating encoder and decoder features to preserve skip-connection alignment. No metadata or explicit conditioning mechanism was included in this baseline. The architecture is summarized in Figure 1.

#### 3.3.2. nnU-Net Baseline

The second baseline was nnU-Net v2, included as a strong self-configuring reference framework for 3D biomedical image segmentation. Unlike the custom 3D U-Net, nnU-Net v2 defines a complete segmentation pipeline that adapts preprocessing, network configuration, training, and inference to the properties of each dataset. This made it suitable for assessing whether FiLM-based metadata conditioning provides additional benefit when the underlying segmentation pipeline is already highly optimized.

For each experimental scenario, the preprocessed NIfTI volumes were converted to the standard nnU-Net dataset structure using a single MRI input channel and the harmonized five-class label space. Three independent nnU-Net datasets were prepared for CHAOS, AMOS, and CHAOS+AMOS, and planning and preprocessing were performed separately for each scenario using the corresponding dataset fingerprint. All baseline nnU-Net models were trained with the 3d_fullres configuration and fold 0. A custom splits_final.json file was used to match the predefined training and validation partitions, ensuring comparability with the other experiments.

### 3.4. FiLM-Conditioned Architectures

The FiLM-conditioned architectures were derived from the corresponding baseline models by adding metadata-dependent feature modulation modules. In these models, the MRI volume remained the main input, while the case-level metadata vector was used to generate scaling and shifting parameters that modulated intermediate feature maps. Therefore, metadata were not concatenated to the image but used as contextual information to adapt the internal representations of the network.

Feature-wise Linear Modulation was implemented as a lightweight affine conditioning mechanism, following the general FiLM framework proposed by Perez et al. [14]. Let $x_{i}\in R^{B\times C\times H\times W\times D}$ be an intermediate feature map, where *B* is the batch size, *C* the number of channels, and *H*, *W*, and *D* the spatial dimensions. Let $m\in R^{B\times M}$ be the metadata vector associated with each case. The FiLM generator $h_{i}$, implemented as a multilayer perceptron, receives m as input and generates two channel-wise modulation vectors, $\gamma_{i}$ and $\beta_{i}$:

(3) $$\left[\gamma_{i},\;\beta_{i}\right]\;=\;h_{i}\left(m\right)$$

where $\gamma_{i},\beta_{i}\in R^{B\times C}$. These vectors are broadcast along the spatial dimensions before being applied to the corresponding feature map.

In this implementation, these vectors modulate the feature maps using a residual scaling form:

(4) $$\mathrm{FiLM}\left(x_{i}|m\right)\;=\;\left(1\;+\;\gamma_{i}\right)⊙x_{i}\;+\;\beta_{i}$$

where ⊙ denotes the Hadamard product. This residual formulation allows the FiLM layer to represent an identity mapping when $\gamma_{i}\;=\;0$ and $\beta_{i}\;=\;0$, which can help preserve the behavior of the baseline feature extractor when no modulation is required.

#### 3.4.1. FiLM Integration in U-Net

The FiLM-conditioned U-Net used the same 3D U-Net backbone described in Section 3.3.1. The main modification was the replacement of selected DoubleConv blocks with FiLMDoubleConv blocks, which first applied the standard convolutional operations and then modulated the resulting feature maps using FiLM. The FiLM generator was implemented as a multilayer perceptron with one hidden layer of 64 units, producing the corresponding $\gamma_{i}$ and $\beta_{i}$ parameters for each modulated feature level.

The FiLM insertion points were scenario-dependent and were selected empirically from a limited set of candidate placements during model development. In CHAOS, FiLM was applied to encoder level 3, the bottleneck, and decoder level 3. In AMOS, FiLM was applied more broadly across encoder, bottleneck, and decoder blocks. In the combined CHAOS+AMOS scenario, modulation was restricted to encoder levels 2 and 3 and the bottleneck, leaving the first encoder level and decoder path unmodulated. Apart from these FiLM blocks and the corresponding metadata input, the model remained identical to the baseline 3D U-Net. Since FiLM placement may influence performance, additional exploratory configurations are analyzed in Section 4.4.3.

#### 3.4.2. FiLM Integration in nnU-Net

FiLM was integrated into nnU-Net v2 by extending the PlainConvUNet backbone generated by the standard nnU-Net planning procedure. The resulting architecture, referred to as PlainConvUNetFiLM, preserved the original encoder–decoder structure, input and output channels, deep supervision configuration, and dataset-dependent architectural parameters. The only architectural modification was the insertion of FiLM modules at selected internal stages of the network. These modules applied channel-wise affine modulation to the corresponding feature maps, using $\gamma_{i}$ and $\beta_{i}$ parameters generated from the case-level metadata vector by a multilayer perceptron with hidden dimension 64.

The FiLM placement was adapted to each experimental scenario following the same general conditioning rationale used in the U-Net experiments, although a direct layer-by-layer correspondence was not possible because the PlainConvUNet backbone is generated by the nnU-Net planning procedure. For CHAOS and AMOS, modulation was applied stage-wise within the PlainConvUNet backbone, whereas in the combined CHAOS+AMOS scenario a more selective configuration was used, mainly conditioning deeper encoder stages and leaving the decoder path unmodulated. To provide metadata during training and inference, a custom nnU-Net trainer retrieved the metadata vector associated with each case from a meta_mapping.npy file and passed it to the network through a meta_override mechanism. Thus, all patches from the same volume were conditioned with the same case-level metadata vector. Apart from the FiLM modules and this metadata-passing mechanism, the standard nnU-Net v2 pipeline was kept unchanged, including the 3d_fullres configuration, fold 0, fixed data split, and harmonized five-class segmentation task.

### 3.5. Training Protocol

All models were trained independently for each experimental scenario using the fixed train/validation/test split described at the beginning of Section 3. Within each scenario, baseline and FiLM-conditioned variants followed the same data partitions and training conditions, so that performance differences could be attributed to the addition of metadata conditioning rather than to differences in data splitting or optimization.

The custom 3D U-Net models were trained using volumetric patches with class-balanced sampling. The loss function combined Dice loss, computed without the background class, with class-weighted cross-entropy, and additional present-only Dice terms were included for selected foreground classes. Data augmentation included class-balanced cropping, flips, 90-degree rotations, affine transformations, Gaussian noise, contrast perturbations, and intensity shifts. In all three experimental scenarios, the same main training hyperparameters were used: a patch size of 128 × 128 × 64, six patches per case, a batch size of 2, the AdamW optimizer, a weight decay of $1\;\times \;10^{-5}$, a peak learning rate of $4\;\times \;10^{-4}$, and a minimum learning rate of $1\;\times \;10^{-6}$. Training was allowed to run for a maximum of 300 epochs, with early stopping after 25 epochs without validation Dice improvement, and the checkpoint with the best validation Dice was selected for test evaluation. During validation and test inference, full-volume predictions were obtained using sliding-window inference with a region of interest of 112 × 112 × 64, an overlap of 0.5, and Gaussian weighting.

The nnU-Net v2 models followed the standard nnU-Net workflow. For each scenario, the corresponding dataset was planned, preprocessed, and trained using the 3d_fullres configuration and fold 0. The internal nnU-Net dataset identifiers were 501 for CHAOS, 502 for AMOS, and 503 for CHAOS+AMOS. A custom splits_final.json file ensured that fold 0 matched the predefined training and validation split. Baseline models used the default nnU-Net trainer, whereas FiLM-conditioned models used custom trainers to pass the case-level metadata vector to the FiLM-compatible network. Specifically, nnUNetTrainerFiLM was used for CHAOS and AMOS, and nnUNetTrainerFiLMV3 was used for the combined CHAOS+AMOS scenario. Apart from this metadata-conditioning mechanism, the remaining nnU-Net pipeline was preserved.

All custom preprocessing, metadata extraction, training, inference, post-processing, metric computation, and statistical analyses were implemented in Python 3.12.13 (Python Software Foundation, Wilmington, DE, USA). The custom 3D U-Net and FiLM-conditioned models were trained using CUDA 12.8 and cuDNN 9.10. Numerical computations and statistical analyses were performed using NumPy 2.0.0 and SciPy 1.16.3. Medical image handling was performed using NiBabel 5.2.1 and SimpleITK 2.5.2, while additional image-processing and machine-learning utilities were implemented using scikit-image 0.25.2 and scikit-learn 1.6.1. The nnU-Net experiments were performed using nnU-Net v2 2.6.4. Training and inference were carried out on an NVIDIA L4 GPU with 23,034 MiB of memory (NVIDIA Corporation, Santa Clara, CA, USA).

### 3.6. Post-Processing and Evaluation Metrics

After inference, each model output was converted into a discrete segmentation mask by assigning each voxel to the class with the highest predicted probability. This direct output was considered the raw prediction. A second version, referred to as the clean prediction, was generated using connected-component-based post-processing.

Post-processing was applied to remove small isolated regions, disconnected false positives, and anatomically implausible components. Connected-component analysis was performed independently for each foreground class using 26-connectivity, retaining the main component and removing smaller disconnected regions. To avoid excessive correction, conservative safeguards were included: when the cleaning step removed an organ completely or produced an implausibly large reduction of the predicted foreground, the original raw prediction for that class was preserved. The same procedure was applied to all models and scenarios.

Segmentation performance was evaluated for both raw and post-processed predictions using the Dice Similarity Coefficient (DSC), Hausdorff Distance at the 95th percentile (HD95), and Average Surface Distance (ASD). These metrics were selected to assess both volumetric overlap and boundary accuracy in 3D abdominal organ segmentation. For a predicted mask *P* and a ground-truth mask *G*, Dice was defined as:

(5) $$\mathrm{DSC}\left(P,\;G\right)\;=\;\frac{2|P\;\cap \;G|}{\left|P|\;+\;|G\right|}$$

Dice was computed independently for each foreground class and then averaged across target classes. HD95 was used to quantify large boundary errors while reducing sensitivity to isolated outliers, whereas ASD measured the average distance between predicted and reference surfaces. Both surface metrics were reported in millimeters using the physical voxel spacing of each volume. Reported values correspond to the mean across foreground classes and test cases. Evaluating both raw and post-processed predictions allowed the analysis to quantify the effect of connected-component cleaning and to distinguish between the intrinsic model output and the final segmentation pipeline.

### 3.7. Statistical Analysis

Statistical analysis was performed on the clean predictions, since these represent the final output of the complete segmentation pipeline after connected-component post-processing. For each dataset and architecture, baseline and FiLM-conditioned models were compared using paired results obtained from the same test cases. For the primary case-level analysis, Dice, HD95, and ASD were computed for each foreground organ and averaged across organs to obtain one value per case. Thus, for a metric *m* and test case *i*, the case-level score was defined as

(6) $$m_{i}\;=\;\frac{1}{\left|O\right|}\sum_{o\in O}m_{i,o},$$

where O denotes the set of foreground organs: liver, right kidney, left kidney, and spleen. The paired difference between FiLM and baseline was then computed as

(7) $$d_{i}\;=\;m_{i}^{\mathrm{FiLM}}\;-\;m_{i}^{\mathrm{Baseline}}.$$

Results were summarized using the median and interquartile range of the case-level scores, together with the median paired difference, denoted as $\tilde{d}$:

(8) $$\tilde{d}\;=\;\mathrm{median}\left(d_{1},\;d_{2},\;\ldots ,\;d_{n}\right).$$

For Dice, positive values of $d_{i}$ indicate improved overlap with FiLM, whereas for HD95 and ASD, negative values indicate improved surface accuracy. Since $\tilde{d}$ was computed from the paired case-wise differences, it does not necessarily equal the difference between the two reported medians.

Since the metric distributions were not assumed to be normal and the comparisons were paired, the Wilcoxon signed-rank test was used to assess whether the median paired difference differed from zero. The null hypothesis was defined as $H_{0}:\tilde{d}=0$. For each comparison, zero differences were discarded, the absolute differences $|d_{i}|$ were ranked, and each rank $R_{i}$ was assigned the sign of the original difference. The positive and negative rank sums were computed as

(9) $$W^{+}\;=\;\sum_{d_{i}>0}R_{i},\;W^{-}\;=\;\sum_{d_{i}<0}R_{i}.$$

For two-sided testing, the Wilcoxon statistic was defined as

(10) $$W\;=\;\min(W^{+},\;W^{-}),$$

and the corresponding *p*-value was obtained from its null distribution. In addition to the primary case-level analysis, Dice was analyzed independently for each foreground organ to assess the anatomical consistency of the FiLM effect. Organ-wise comparisons were also performed using paired Wilcoxon signed-rank tests. Since multiple organ-wise comparisons were performed within each dataset–architecture setting, the resulting *p*-values were adjusted using the Holm–Bonferroni method to control the family-wise error rate. In this procedure, the unadjusted *p*-values are sorted in ascending order and compared sequentially with increasingly less stringent significance thresholds. Adjusted *p*-values were capped at 1. A significance level of α=0.05 was used throughout the analysis.

## 4. Results

This section reports the quantitative evaluation performed in the three experimental scenarios. The baseline and FiLM-conditioned versions of both 3D U-Net and nnU-Net v2 were compared using Dice, HD95, and ASD, considering both raw model outputs and connected-component post-processed predictions. Results are first presented separately for each scenario, followed by a compact cross-scenario analysis. Additional analyses then examine the effect of FiLM conditioning, the sensitivity to the FiLM configuration, the statistical significance of the observed differences, the computational overhead introduced by FiLM, and the impact of post-processing.

### 4.1. CHAOS Dataset

The first experimental scenario evaluated the four model configurations on the CHAOS MRI dataset. In this scenario, FiLM-conditioned models used sequence metadata as conditioning information.

The quantitative results on the CHAOS dataset are reported in Table 3. On CHAOS, nnU-Net achieved the highest clean Dice value, with 0.9257. In the 3D U-Net models, FiLM slightly improved clean performance, increasing Dice from 0.9005 to 0.9056 and reducing HD95 from 9.4780 mm to 8.2495 mm and ASD from 2.1367 mm to 1.9364 mm. In contrast, nnU-Net and nnU-Net + FiLM produced very similar clean results, indicating that sequence-based FiLM conditioning had little effect on nnU-Net in this scenario.

### 4.2. AMOS Dataset

The second experimental scenario evaluated the four model configurations on the AMOS MRI subset. In this scenario, FiLM-conditioned models used intensity metadata as conditioning information.

Quantitative findings for the AMOS scenario are summarized in Table 4. Baseline nnU-Net achieved the highest clean Dice value, with 0.9262. In the 3D U-Net models, FiLM improved clean performance, increasing Dice from 0.9054 to 0.9208 and reducing HD95 from 9.5000 mm to 7.9490 mm and ASD from 2.3900 mm to 1.5287 mm. This made U-Net + FiLM the best-performing model in terms of clean surface-distance metrics. In contrast, nnU-Net + FiLM reduced clean Dice compared with the nnU-Net baseline, from 0.9262 to 0.8997, while producing only mixed changes in the surface metrics.

### 4.3. Combined CHAOS and AMOS Datasets

The third experimental scenario evaluated the four model configurations on the combined CHAOS+AMOS dataset, representing the multi-domain setting of the study. In this scenario, FiLM-conditioned models used foreground intensity metadata as conditioning information.

Results for the multi-domain CHAOS+AMOS scenario are reported in Table 5. Baseline nnU-Net achieved the best clean performance across all metrics, with a Dice of 0.9346, HD95 of 6.1100 mm, and ASD of 1.1500 mm. In the 3D U-Net models, FiLM produced the largest improvement observed for this architecture, increasing clean Dice from 0.8868 to 0.9083 and reducing clean HD95 from 26.2700 mm to 19.6100 mm and clean ASD from 7.6300 mm to 3.7800 mm. In contrast, nnU-Net + FiLM obtained lower clean performance than the nnU-Net baseline, although it remained more accurate than the U-Net-based models in this multi-domain setting.

### 4.4. Cross-Scenario Visual Summary

**Figure 2:**
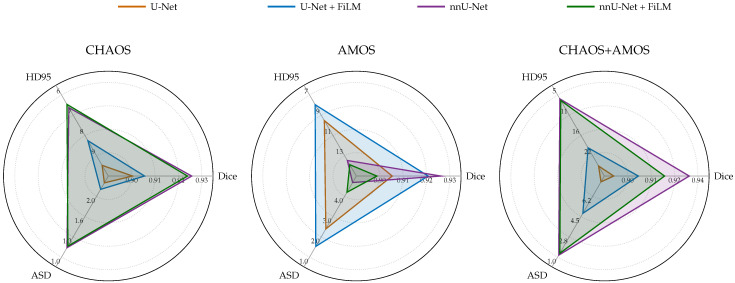
Radar summary of clean segmentation performance across the three experimental scenarios: CHAOS, AMOS, and CHAOS+AMOS. Each radar plot compares the four evaluated model configurations using Dice, HD95, and ASD after connected-component post-processing. Higher Dice values indicate better overlap, whereas lower HD95 and ASD values indicate better boundary accuracy. Axis ranges were adjusted independently for each scenario to improve readability; therefore, comparisons should be interpreted within each panel rather than across panels.

Figure 2 provides a compact visual overview of the clean segmentation performance across the three experimental scenarios and the four evaluated model configurations.

#### 4.4.1. Effect of FiLM

**Figure 3:**
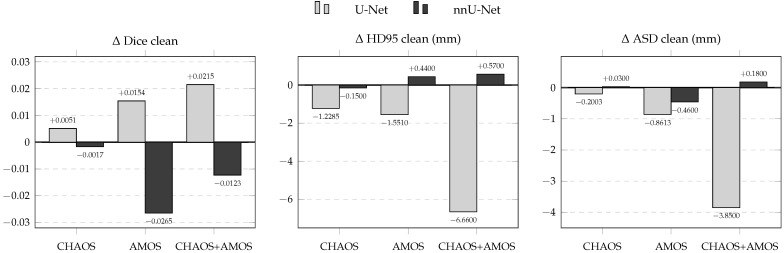
Quantitative summary of the effect of FiLM conditioning on clean segmentation performance (Dice, HD95, and ASD) across both architectures and all scenarios. Delta values are computed as FiLM minus baseline, where positive Dice deltas and negative HD95/ASD deltas signify performance gains.

To assess the effect of FiLM conditioning, delta values were computed from the clean predictions by subtracting each baseline result from its corresponding FiLM-conditioned model. Positive values in Dice indicate improved overlap after FiLM conditioning, whereas negative values in HD95 and ASD indicate reduced surface error. The resulting deltas are summarized in Figure 3.

#### 4.4.2. Effect of Post-Processing

**Figure 4:**
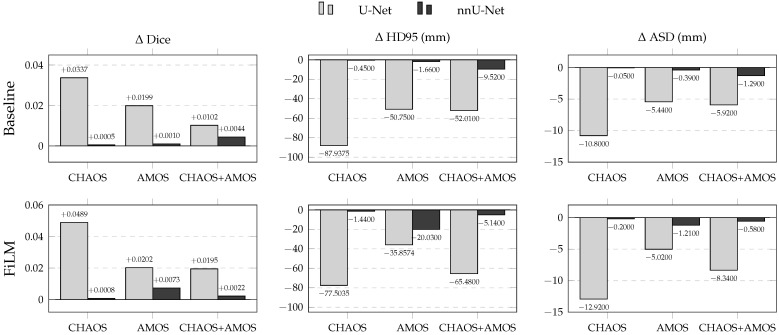
Impact of connected-component post-processing on segmentation performance across models and datasets. Delta values were computed as clean minus raw for Dice, HD95, and ASD.

The effect of connected-component post-processing was assessed by computing the difference between clean and raw predictions for each model. Delta values were calculated as clean minus raw. Therefore, positive values in Dice indicate improved overlap after post-processing, whereas negative values in HD95 and ASD indicate reduced surface error. The resulting deltas are summarized in Figure 4.

#### 4.4.3. Exploratory FiLM Configuration Analysis

To complement the main FiLM analysis, a small set of representative exploratory configurations was included to illustrate the sensitivity of the method to metadata selection and modulation design. These exploratory tests were restricted to the 3D U-Net architecture because the choice of metadata and FiLM layer placement was mainly optimized during the development of this model. The most relevant conditioning strategies were then transferred to nnU-Net, where additional experimentation was more limited due to the greater complexity of modifying the nnU-Net pipeline. Table 6 summarizes the selected exploratory U-Net configurations.

#### 4.4.4. Statistical Comparison of FiLM Effects

The paired statistical analysis was performed on the clean predictions to assess whether the differences between baseline and FiLM-conditioned models were consistent at the case level. Table 7 reports the median and IQR for each metric, together with the median paired difference ($\tilde{d}$) and the Wilcoxon signed-rank *p*-value. FiLM showed favourable case-level trends for U-Net, with increased Dice and reduced surface distances in most scenarios. However, statistical significance was only reached for ASD in CHAOS and CHAOS+AMOS. In contrast, nnU-Net did not show statistically significant improvements with FiLM in any case-level comparison.

To further assess the anatomical distribution of these effects, an organ-wise Dice analysis was performed and is represented in Table 8. Since multiple organ-wise comparisons were involved, Holm–Bonferroni-adjusted *p*-values are also reported. The organ-wise results showed small and anatomically variable Dice differences. In U-Net, several organs presented positive median differences, but none remained significant after correction. In nnU-Net, only the AMOS liver comparison remained significant after correction; however, this isolated finding was not accompanied by consistent improvements across organs or datasets.

#### 4.4.5. Computational Cost

Computational cost was assessed in the combined CHAOS+AMOS scenario to quantify the overhead introduced by FiLM conditioning. All measurements were obtained on the same NVIDIA L4 GPU. Table 9 reports the number of trainable parameters, the percentage of parameters corresponding to the FiLM modules, model size, forward-pass time per patch, forward-plus-backward time per patch, full-volume inference time, and peak GPU memory allocated during inference. Forward-plus-backward time was included as an estimate of the relative cost of a training-like step, whereas inference time was measured using the corresponding sliding-window inference pipeline for each model.

## 5. Discussion

This section discusses the main findings of the comparative evaluation between baseline and FiLM-conditioned segmentation models. The analysis focuses on the architecture-dependent effect of FiLM, model behavior under acquisition- and dataset-related heterogeneity, the role of connected-component post-processing, and the statistical and computational evidence supporting the observed trends.

### 5.1. Architecture-Dependent Effect of FiLM: Quantitative and Statistical Evidence

The main finding of this study is that the effect of FiLM conditioning was architecture-dependent. In the standard 3D U-Net, FiLM produced favourable quantitative changes in the clean predictions, especially in the AMOS and CHAOS+AMOS scenarios. In contrast, the same conditioning strategy did not provide a consistent benefit when integrated into nnU-Net v2. This indicates that the effect of metadata conditioning depends not only on the availability of auxiliary information, but also on the adaptability already present in the segmentation framework.

In U-Net, the largest quantitative improvement was observed in the combined CHAOS+AMOS scenario, as reported in Table 5. In this setting, FiLM increased clean Dice from 0.8868 to 0.9083 and reduced clean HD95 and ASD by 6.66mm and 3.85mm, respectively. This behaviour suggests that FiLM may help a generic encoder–decoder architecture adapt its intermediate representations when acquisition- and dataset-related heterogeneity increases. However, the paired statistical analysis showed that these improvements should be interpreted as favourable trends rather than uniform statistically significant gains. As shown in Table 7, statistical significance at case level was reached only for ASD in CHAOS and CHAOS+AMOS, while Dice and HD95 did not show significant improvements across all scenarios.

For nnU-Net, FiLM did not lead to statistically supported improvements at case level. Although some surface-distance values changed slightly in specific scenarios, the baseline nnU-Net remained the strongest overall model and the FiLM-conditioned variant did not show a robust advantage. A plausible explanation is that nnU-Net already incorporates a high degree of adaptation through its self-configuring pipeline, including dataset-specific preprocessing, normalization, target spacing, patch size, network configuration, training strategy, and inference. In this context, additional feature modulation may become redundant or may interfere with representations that are already well adapted by the baseline pipeline. However, this interpretation should be regarded as a qualitative hypothesis supported by the observed quantitative and statistical trends, rather than as a causal mechanism directly demonstrated by this study. A mechanistic validation would require additional analyses of internal activations, learned FiLM parameters, feature representations, and targeted ablation experiments within the nnU-Net pipeline.

The organ-wise Dice analysis, summarized in Table 8, further supports a cautious interpretation. In U-Net, several organs showed positive median differences with FiLM, but none remained statistically significant after Holm–Bonferroni correction. In nnU-Net, only the AMOS liver comparison remained significant after correction, but this isolated result was not accompanied by consistent improvements across organs, datasets, or metrics. Therefore, the statistical analysis does not support a uniform organ-specific benefit of FiLM, but rather suggests that its effect is small, variable, and dependent on both architecture and scenario.

**Figure 5:**
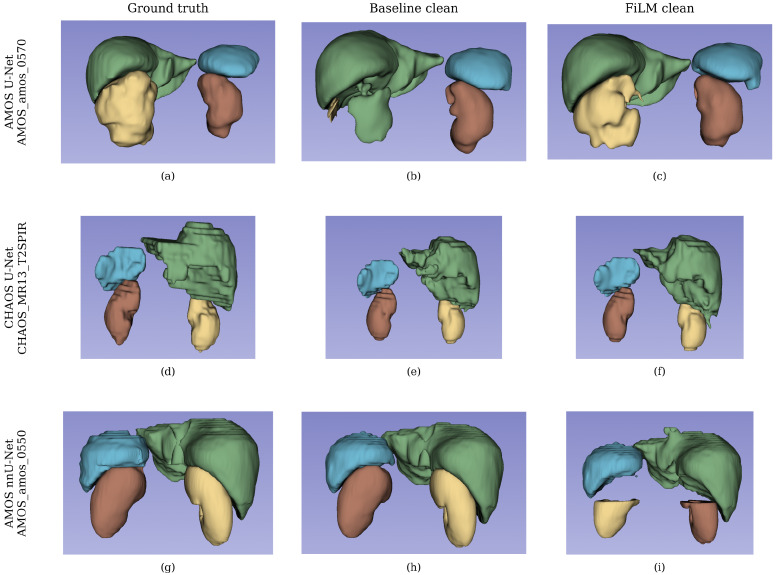
Representative examples of the architecture-dependent effect of FiLM conditioning on clean 3D abdominal organ segmentations. Rows correspond to AMOS U-Net, CHAOS U-Net, and AMOS nnU-Net, respectively. Columns show the ground-truth segmentation, the clean prediction obtained with the corresponding baseline model, and the clean prediction obtained with the FiLM-conditioned model. Panels (**a**–**c**) correspond to AMOS_amos_0570, panels (**d**–**f**) to CHAOS_MR13_T2SPIR, and panels (**g**–**i**) to AMOS_amos_0550. Organ colors are consistent across panels: liver is shown in green, right kidney in yellow, left kidney in brown, and spleen in light blue.

The representative examples shown in Figure 5 are consistent with this interpretation. In the U-Net examples, FiLM improves the anatomical consistency of the clean predictions, particularly in the AMOS case, whereas its effect is more moderate in CHAOS. In contrast, the nnU-Net example illustrates that the baseline prediction is already close to the reference segmentation, while the FiLM-conditioned version introduces a visible degradation.

Overall, these findings suggest that FiLM can be useful as a lightweight conditioning mechanism for a generic 3D U-Net, particularly under heterogeneous acquisition conditions. Nevertheless, the statistical results indicate that this benefit is not uniform across metrics or organs. For nnU-Net, the evaluated FiLM configurations did not provide additional robust value over the self-configuring baseline. Therefore, FiLM should be interpreted as an architecture-dependent strategy rather than as a universally beneficial modification.

### 5.2. Interpretation of FiLM Configuration Sensitivity

The exploratory FiLM configurations reported in Table 6 indicate that the effect of metadata conditioning depends on the specific modulation design. In particular, FiLM performance was influenced by the selected metadata, the insertion points within the network, and the underlying architecture. This supports the interpretation that FiLM should not be regarded as a plug-and-play improvement, but as a context-dependent conditioning mechanism whose effectiveness must be validated for each segmentation setting.

For this reason, the FiLM placements used in the main experiments were selected empirically from a limited set of candidate configurations during model development. Localized FiLM insertion provides a more constrained form of conditioning, whereas applying FiLM across several encoder, bottleneck, or decoder stages increases the strength of metadata-driven adaptation but may also increase the risk of over-conditioning, particularly in small datasets. Therefore, the purpose of this analysis was not to provide an exhaustive ablation study of all possible FiLM designs, but to assess whether alternative reasonable configurations led to similar or markedly different behaviour. The observed sensitivity reinforces the need to consider FiLM placement, metadata type, and baseline architecture when applying metadata conditioning to heterogeneous abdominal MRI segmentation.

### 5.3. Multi-Domain Robustness and Acquisition Variability

The combined CHAOS+AMOS experiment is useful for interpreting how metadata conditioning behaves when segmentation is performed across data from different MRI sources. Rather than providing a uniformly more robust model, FiLM showed an architecture-dependent response: it was more helpful for the generic U-Net backbone, but it did not improve the already self-configuring nnU-Net pipeline. This suggests that metadata conditioning may partially support robustness under dataset-related variability, but only when the baseline architecture does not already include strong mechanisms for adapting preprocessing, normalization, training, and inference to the data.

Therefore, the multi-domain results should not be interpreted as evidence that FiLM generally solves acquisition variability. Instead, they indicate that the value of metadata conditioning depends on how much variability remains unaccounted for by the segmentation pipeline itself. In this sense, FiLM appears more appropriate as a lightweight adaptation mechanism for simpler architectures than as a direct robustness enhancement for highly optimized frameworks such as nnU-Net.

### 5.4. Role of Post-Processing Across Segmentation Architectures

Connected-component post-processing had a markedly different impact depending on the architecture. Its effect was more evident in the U-Net-based models, where raw predictions were more frequently affected by isolated false positives and disconnected components. In these cases, post-processing mainly improved structural consistency and surface-distance metrics by removing anatomically implausible regions. In contrast, nnU-Net showed much smaller raw-to-clean changes, suggesting that its predictions were already more spatially coherent before post-processing.

**Figure 6:**
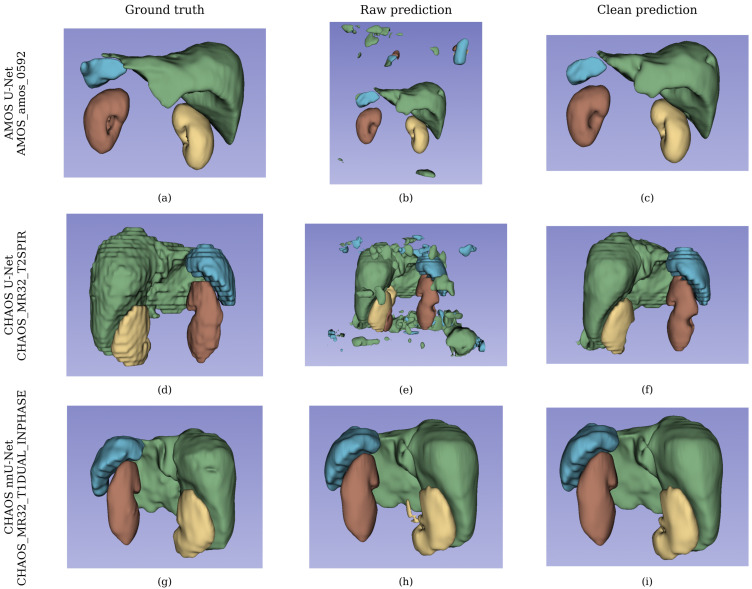
Representative examples of the effect of connected-component post-processing on 3D abdominal organ segmentations. Rows correspond to AMOS U-Net, CHAOS U-Net, and CHAOS nnU-Net, respectively. Columns show the ground-truth segmentation, the raw model prediction, and the clean prediction obtained after post-processing. Panels (**a**–**c**) correspond to AMOS_amos_0592, panels (**d**–**f**) to CHAOS_MR32_T2SPIR, and panels (**g**–**i**) to CHAOS_MR32_T1DUAL_INPHASE. Organ colors are consistent across panels: liver is shown in green, right kidney in yellow, left kidney in brown, and spleen in light blue.

This pattern is illustrated in Figure 6. In the U-Net examples, post-processing visibly removes disconnected components and produces cleaner organ masks, whereas in the nnU-Net example the raw prediction is already close to the post-processed output. Therefore, post-processing should be interpreted as an important refinement step for U-Net-based models, but as a comparatively minor correction for nnU-Net, whose baseline pipeline already produces more stable raw segmentations.

### 5.5. Computational Cost and Practical Relevance

The computational analysis showed that FiLM introduced only a small overhead with respect to each baseline architecture. As reported in Table 9, the FiLM-conditioned U-Net increased the number of trainable parameters from 5.60 M to 5.66 M, with FiLM modules representing 1.05% of the total parameters. Similarly, nnU-Net + FiLM increased the parameter count from 30.71 M to 30.85 M, with FiLM accounting for 0.47% of the model parameters. These results confirm that the proposed conditioning mechanism is lightweight in terms of model size and parameter cost.

From a practical perspective, the relevance of this overhead depends on the baseline architecture. In U-Net, FiLM produced favourable quantitative trends while preserving a substantially smaller model than nnU-Net. Although U-Net + FiLM did not surpass the baseline nnU-Net, it reduced part of the performance gap in the combined CHAOS+AMOS setting with a lower computational footprint, which may be relevant when memory, inference time, hardware availability, or implementation simplicity are important constraints. In contrast, the low overhead of FiLM did not translate into a clear advantage for nnU-Net, where the conditioned variant did not provide robust performance gains. Therefore, the practical value of FiLM should be assessed as a balance between added complexity and measurable improvement, which in this study was more favourable for the generic U-Net than for the self-configuring nnU-Net framework.

### 5.6. Implications for Biomedical Imaging Systems

From the perspective of biomedical imaging systems, the results highlight the importance of considering the acquisition context of MRI data when designing automatic segmentation models. MRI volumes are generated by specific acquisition systems and protocols, and their appearance can vary according to sequence type, spatial resolution, contrast properties, reconstruction process, and intensity distribution. These factors are not only technical characteristics of the input data, but also potential sources of variability that can affect model reliability when public benchmarks or multi-source datasets are combined.

In this context, acquisition- or image-derived metadata can provide a lightweight way to expose part of this variability to the segmentation model. However, the results also show that metadata conditioning should not be applied as a generic solution. Its practical value depends on the information contained in the available metadata, the heterogeneity of the imaging data, the preprocessing strategy, and the adaptability of the segmentation architecture. Therefore, metadata-aware segmentation may be useful for biomedical imaging pipelines, particularly when acquisition variability is relevant and the baseline model does not already include strong mechanisms for handling such variability.

### 5.7. Limitations

This study has several limitations. First, the experiments were based on two public abdominal MRI datasets, CHAOS and AMOS, with relatively limited test sets. Although these datasets provide useful benchmarks for evaluating segmentation under acquisition- and dataset-related variability, validation on larger external and multi-center cohorts would be necessary to confirm the generality of the findings. In addition, the available acquisition information was not homogeneous across datasets, which limited the possibility of explicitly analyzing the effect of scanner vendor, magnetic field strength, acquisition parameters, reconstruction settings, or detailed protocol information.

Second, the metadata variables and FiLM insertion strategies were manually selected and scenario-dependent. Although a limited set of exploratory configurations was evaluated, this analysis was not intended as an exhaustive ablation study of all possible metadata types, insertion points, generator architectures, or conditioning mechanisms. Other metadata combinations, automatically selected descriptors, or alternative modulation designs could lead to different results, particularly if more complete and homogeneous acquisition information were available. Therefore, the conclusions should be interpreted in relation to the specific FiLM implementations evaluated in this work.

Third, the comparison was performed using a fixed train/validation/test split to ensure direct paired evaluation between baseline and FiLM-conditioned models. Although paired statistical testing was included, the limited number of test cases and the absence of repeated splits or cross-validation restrict the assessment of result stability. Future work should include repeated partitions, cross-validation, or external test cohorts to better quantify the robustness of the observed trends.

Fourth, the present evaluation was restricted to abdominal MRI and to four abdominal organs. Therefore, the findings cannot be assumed to directly generalize to other imaging modalities, anatomical regions, segmentation targets, or clinical tasks. Future work should evaluate whether FiLM-based metadata conditioning provides similar benefits in other medical imaging modalities, such as CT or ultrasound, as well as in larger multi-center datasets with more complete and harmonized acquisition metadata.

Finally, the computational analysis was performed on a single hardware configuration and focused on the combined CHAOS+AMOS scenario. Inference time, memory usage, and training overhead may vary depending on the implementation, GPU, patch size, batch size, and inference strategy. Moreover, the evaluation was based on quantitative metrics and representative visual examples, but no prospective clinical validation was performed. Further assessment would therefore be required to determine the practical impact of metadata-conditioned segmentation models in real biomedical imaging workflows.

## 6. Conclusions

This work evaluated Feature-wise Linear Modulation as a metadata-conditioning strategy for 3D abdominal organ segmentation in MRI. A standard 3D U-Net and nnU-Net v2 were compared in baseline and FiLM-conditioned variants across three scenarios: CHAOS, AMOS, and the combined CHAOS+AMOS setting. The study was designed to assess whether acquisition- or image-derived metadata can improve segmentation under heterogeneous MRI conditions, and whether this effect depends on the underlying segmentation framework.

The results indicate that the effect of FiLM was clearly architecture-dependent. In the standard 3D U-Net, FiLM produced favourable quantitative trends, particularly in the AMOS and CHAOS+AMOS scenarios. In the combined setting, U-Net + FiLM increased clean Dice from 0.8868 to 0.9083 and reduced clean HD95 and ASD by 6.66mm and 3.85mm, respectively. However, the statistical analysis showed that these improvements were not uniform across all metrics or organs. At case level, statistical significance was reached only for ASD in CHAOS and CHAOS+AMOS, while the organ-wise Dice analysis did not support a consistent anatomical benefit after correction for multiple comparisons. Therefore, FiLM should be interpreted as providing favourable but scenario-dependent improvements in U-Net, rather than a uniformly significant gain.

In contrast, the evaluated FiLM configurations did not provide a robust advantage when integrated into nnU-Net v2. The baseline nnU-Net remained the strongest overall model, especially in the combined CHAOS+AMOS scenario, where it achieved the best clean Dice, HD95, and ASD values. This suggests that the self-configuring nnU-Net pipeline may already account for part of the variability that FiLM aims to address through its dataset-specific preprocessing, planning, training, and inference strategies. In this context, additional feature modulation may be redundant or may interfere with representations that are already well adapted by the baseline framework.

The analysis also showed that FiLM is sensitive to the selected metadata, insertion points, modulation design, and baseline architecture. Thus, metadata conditioning should not be considered a plug-and-play improvement, but a design-dependent mechanism that requires validation for each segmentation setting. Similarly, connected-component post-processing had an architecture-dependent effect: it was more relevant for U-Net-based models, where it helped remove disconnected components and improve surface consistency, whereas nnU-Net produced more stable raw predictions with comparatively smaller raw-to-clean changes.

From a practical perspective, FiLM introduced only a small computational overhead. This makes U-Net + FiLM a potentially relevant lightweight alternative when memory, inference time, hardware availability, or implementation simplicity are important constraints. Nevertheless, U-Net + FiLM did not surpass the baseline nnU-Net, and in nnU-Net the low overhead of FiLM did not translate into robust performance gains. Therefore, the practical value of metadata conditioning should be assessed as a balance between additional complexity and measurable improvement.

Overall, this study supports the idea that metadata conditioning can be useful for reinforcing generic segmentation architectures under heterogeneous abdominal MRI conditions, but its benefit is not universal. Its effectiveness depends on the available metadata, the modulation design, the degree of acquisition-related variability, and the adaptability of the segmentation framework. For biomedical imaging systems, these findings highlight the importance of considering acquisition context when designing segmentation models, while also showing that metadata-aware approaches should be adapted to the characteristics of each pipeline rather than applied as general-purpose modifications.

Future work should validate these findings on larger external and multi-center MRI cohorts, ideally including more complete and homogeneous acquisition information, such as scanner vendor, magnetic field strength, acquisition parameters, reconstruction settings, and detailed protocol descriptors. Further studies should also investigate automatic metadata selection, alternative conditioning mechanisms, repeated splits or cross-validation, and prospective clinical evaluation. In self-configuring frameworks such as nnU-Net, future approaches could explore incorporating metadata into fingerprinting, planning, preprocessing, normalization, augmentation, or adaptation stages, rather than adding feature modulation only as an external architectural modification.

## Figures and Tables

**Table 1:** Summary of the dataset composition and fixed 70/15/15 train/validation/test split for each experimental scenario. In CHAOS, splits were performed at subject level to avoid leakage between sequences from the same subject.

Scenario	Total Cases	Train	Val.	Test	Notes
CHAOS MRI	60	42	9	9	20 subjects, 3 MRI sequences per subject
AMOS MRI	60	42	9	9	MRI cases with available target-organ labels
CHAOS+AMOS	120	84	18	18	Combined multi-domain MRI scenario

**Table 2:** Summary of metadata types and specific variables used for conditioning the FiLM-based architectures in each experimental scenario. Variables were selected to capture contextual information from acquisition protocols or image-derived intensity characteristics.

Scenario	Metadata Type	Metadata Variables
CHAOS	Sequence	T1 in-phase, T1 out-of-phase, T2-SPIR
AMOS	Intensity	${\mathrm{mean}}_{z}$, ${\mathrm{std}}_{z}$, $p05_{z}$, $p95_{z}$
CHAOS+AMOS	Foreground intensity	$p25_{z}$, $p75_{z}$, $\mathrm{contrast}\text{\_}{\mathrm{ratio}}_{z}$, $\mathrm{mean}\text{\_}{\mathrm{fg}}_{z}$, $\mathrm{skew}\text{\_}{\mathrm{fg}}_{z}$

**Table 3:** Quantitative segmentation results on the CHAOS dataset. Dice, HD95, and ASD are reported for raw predictions and clean predictions after connected-component post-processing. HD95 and ASD are expressed in millimeters.

Model	Dice Raw	Dice Clean	HD95 Raw (mm)	HD95 Clean (mm)	ASD Raw (mm)	ASD Clean (mm)
U-Net	0.8668	0.9005	97.4155	9.4780	12.9400	2.1367
U-Net + FiLM	0.8567	0.9056	85.7530	8.2495	14.8590	1.9364
nnU-Net	0.9252	0.9257	7.0600	6.6100	1.2300	1.1800
nnU-Net + FiLM	0.9232	0.9240	7.9000	6.4600	1.4100	1.2100

**Table 4:** Quantitative segmentation results on the AMOS dataset. Dice, HD95, and ASD are reported for raw predictions and clean predictions after connected-component post-processing. HD95 and ASD are expressed in millimeters.

Model	Dice Raw	Dice Clean	HD95 Raw (mm)	HD95 Clean (mm)	ASD Raw (mm)	ASD Clean (mm)
U-Net	0.8855	0.9054	60.2400	9.5000	7.8300	2.3900
U-Net + FiLM	0.9006	0.9208	43.8064	7.9490	6.5449	1.5287
nnU-Net	0.9252	0.9262	15.1000	13.4400	5.0600	4.6700
nnU-Net + FiLM	0.8925	0.8997	33.9100	13.8800	5.4200	4.2100

**Table 5:** Quantitative segmentation results on the combined CHAOS+AMOS dataset. Dice, HD95, and ASD are reported for raw predictions and clean predictions after connected-component post-processing. HD95 and ASD are expressed in millimeters.

Model	Dice Raw	Dice Clean	HD95 Raw (mm)	HD95 Clean (mm)	ASD Raw (mm)	ASD Clean (mm)
U-Net	0.8766	0.8868	78.2800	26.2700	13.5500	7.6300
U-Net + FiLM	0.8889	0.9083	85.0900	19.6100	12.1200	3.7800
nnU-Net	0.9302	0.9346	15.6300	6.1100	2.4400	1.1500
nnU-Net + FiLM	0.9201	0.9223	11.8200	6.6800	1.9100	1.3300

**Table 6:** Representative exploratory FiLM configurations evaluated with the 3D U-Net architecture. Results are reported for clean predictions after connected-component post-processing. HD95 and ASD are expressed in millimeters.

Scenario	Metadata	FiLM Configuration	Dice Clean	HD95 Clean (mm)	ASD Clean (mm)
CHAOS	Global intensity descriptors ($mean_{z}$, $std_{z}$, $p05_{z}$, $p95_{z}$)	Deep FiLM modulation at encoder level 3, bottleneck, and decoder; residual clamped FiLM	0.8751	12.9663	2.7716
AMOS	Spacing and global intensity descriptors	FiLM modulation in all U-Net blocks, including encoder, bottleneck, and decoder; residual clamped FiLM	0.8800	15.8228	8.7489
CHAOS+AMOS	Domain and sequence one-hot metadata	FiLM modulation in all U-Net blocks, including encoder, bottleneck, and decoder	0.8908	27.2187	5.3906

**Table 7:** Paired case-level statistical analysis comparing baseline and FiLM-conditioned models using clean predictions. Values are reported as median [IQR]. $\tilde{d}$ denotes the median of the paired case-wise differences between FiLM and baseline. HD95 and ASD are expressed in millimeters.

Dataset	Arch.	Metric	*n*	Baseline Median [IQR]	FiLM Median [IQR]	$\tilde{d}$	Wilcoxon *p*
CHAOS	U-Net	Dice	9	0.8949 [0.8810–0.9276]	0.9071 [0.8867–0.9287]	+0.0028	0.1250
CHAOS	U-Net	HD95 (mm)	9	7.08 [5.44–14.16]	5.88 [5.40–12.64]	−0.53	0.2500
CHAOS	U-Net	ASD (mm)	9	2.03 [1.54–2.79]	1.63 [1.48–2.38]	−0.14	0.0391
CHAOS	nnU-Net	Dice	9	0.9291 [0.9145–0.9337]	0.9207 [0.9151–0.9317]	−0.0020	0.3594
CHAOS	nnU-Net	HD95 (mm)	9	6.31 [5.88–6.36]	6.39 [6.21–7.27]	−0.01	0.9102
CHAOS	nnU-Net	ASD (mm)	9	1.10 [1.07–1.19]	1.26 [1.09–1.31]	+0.03	0.3125
AMOS	U-Net	Dice	9	0.9368 [0.9034–0.9437]	0.9444 [0.8934–0.9537]	+0.0042	0.2031
AMOS	U-Net	HD95 (mm)	9	8.53 [5.03–9.12]	6.07 [5.20–10.59]	−0.02	0.9102
AMOS	U-Net	ASD (mm)	9	1.23 [1.07–1.79]	1.07 [0.96–2.08]	−0.03	0.5469
AMOS	nnU-Net	Dice	9	0.9596 [0.9236–0.9729]	0.9686 [0.9232–0.9730]	−0.0021	0.3008
AMOS	nnU-Net	HD95 (mm)	9	3.58 [2.65–8.75]	3.19 [3.13–13.01]	+0.52	0.3008
AMOS	nnU-Net	ASD (mm)	9	0.69 [0.58–1.33]	0.65 [0.53–1.42]	+0.04	0.4258
CHAOS+AMOS	U-Net	Dice	18	0.9058 [0.8433–0.9380]	0.9128 [0.8948–0.9414]	+0.0071	0.0987
CHAOS+AMOS	U-Net	HD95 (mm)	18	17.50 [5.47–32.57]	6.53 [5.44–35.85]	−0.57	0.1084
CHAOS+AMOS	U-Net	ASD (mm)	18	2.90 [1.74–7.94]	2.11 [1.28–4.30]	−0.55	0.0432
CHAOS+AMOS	nnU-Net	Dice	18	0.9415 [0.9189–0.9535]	0.9369 [0.9129–0.9564]	+0.0020	0.9323
CHAOS+AMOS	nnU-Net	HD95 (mm)	18	4.55 [3.44–5.62]	5.09 [3.36–6.45]	−0.15	0.8650
CHAOS+AMOS	nnU-Net	ASD (mm)	18	0.97 [0.78–1.19]	0.98 [0.78–1.14]	−0.04	0.6947

**Table 8:** Organ-wise Dice analysis comparing baseline and FiLM-conditioned models using clean predictions. Values are reported as median [IQR]. ${\tilde{d}}_{\mathrm{Dice}}$ denotes the median of the paired case-wise Dice differences between FiLM and baseline. Wilcoxon *p*-values were adjusted using the Holm–Bonferroni method within each dataset–architecture setting.

Dataset	Arch.	Organ	*n*	Baseline Dice	FiLM Dice	${\tilde{d}}_{\mathrm{Dice}}$	*p*	$p_{\mathrm{Holm}}$
CHAOS	U-Net	Liver	9	0.9140 [0.9085–0.9256]	0.9160 [0.9066–0.9188]	−0.0019	0.4258	0.8516
CHAOS	U-Net	Right kidney	9	0.8972 [0.8312–0.9382]	0.9044 [0.8180–0.9435]	+0.0041	0.4961	0.8516
CHAOS	U-Net	Left kidney	9	0.9149 [0.8887–0.9411]	0.9166 [0.9031–0.9387]	+0.0068	0.1641	0.5156
CHAOS	U-Net	Spleen	9	0.9038 [0.8864–0.9176]	0.9106 [0.9028–0.9382]	+0.0087	0.1289	0.5156
CHAOS	nnU-Net	Liver	9	0.9430 [0.9164–0.9551]	0.9410 [0.9363–0.9532]	+0.0003	0.8203	1.0000
CHAOS	nnU-Net	Right kidney	9	0.9313 [0.9032–0.9388]	0.9177 [0.8901–0.9265]	−0.0071	0.0195	0.0781
CHAOS	nnU-Net	Left kidney	9	0.9344 [0.9168–0.9407]	0.9274 [0.9062–0.9397]	+0.0011	1.0000	1.0000
CHAOS	nnU-Net	Spleen	9	0.9142 [0.9059–0.9191]	0.9142 [0.9110–0.9206]	+0.0011	0.0781	0.2344
AMOS	U-Net	Liver	9	0.9697 [0.9648–0.9766]	0.9679 [0.9594–0.9681]	−0.0052	0.1289	0.5156
AMOS	U-Net	Right kidney	9	0.9151 [0.8899–0.9429]	0.9303 [0.9207–0.9547]	+0.0031	0.3008	0.6094
AMOS	U-Net	Left kidney	9	0.9447 [0.9144–0.9558]	0.9423 [0.9099–0.9529]	−0.0034	0.7344	0.7344
AMOS	U-Net	Spleen	9	0.9388 [0.8606–0.9559]	0.9316 [0.8786–0.9581]	+0.0092	0.2031	0.6094
AMOS	nnU-Net	Liver	9	0.9784 [0.9726–0.9795]	0.9818 [0.9796–0.9830]	+0.0023	0.0078	0.0312
AMOS	nnU-Net	Right kidney	9	0.9648 [0.9482–0.9725]	0.9399 [0.8663–0.9628]	−0.0052	0.0742	0.2227
AMOS	nnU-Net	Left kidney	9	0.9642 [0.9352–0.9676]	0.9647 [0.9213–0.9659]	−0.0003	0.2500	0.5000
AMOS	nnU-Net	Spleen	9	0.9672 [0.9394–0.9813]	0.9692 [0.9434–0.9809]	−0.0014	0.3594	0.5000
CHAOS+AMOS	U-Net	Liver	18	0.9545 [0.9156–0.9691]	0.9536 [0.9288–0.9718]	+0.0019	0.0385	0.1540
CHAOS+AMOS	U-Net	Right kidney	18	0.9283 [0.8822–0.9420]	0.9354 [0.8935–0.9494]	+0.0044	0.1964	0.4621
CHAOS+AMOS	U-Net	Left kidney	18	0.9344 [0.9125–0.9457]	0.9377 [0.9132–0.9459]	+0.0014	0.1815	0.4621
CHAOS+AMOS	U-Net	Spleen	18	0.9160 [0.7911–0.9353]	0.9076 [0.8829–0.9337]	+0.0077	0.1540	0.4621
CHAOS+AMOS	nnU-Net	Liver	18	0.9622 [0.9433–0.9662]	0.9589 [0.9323–0.9689]	+0.0010	0.4330	1.0000
CHAOS+AMOS	nnU-Net	Right kidney	18	0.9408 [0.9080–0.9544]	0.9437 [0.8934–0.9542]	−0.0023	0.6705	1.0000
CHAOS+AMOS	nnU-Net	Left kidney	18	0.9468 [0.9185–0.9510]	0.9435 [0.9228–0.9570]	+0.0015	0.3692	1.0000
CHAOS+AMOS	nnU-Net	Spleen	18	0.9296 [0.9207–0.9585]	0.9290 [0.8962–0.9533]	+0.0013	0.4951	1.0000

**Table 9:** Computational cost of the evaluated models in the combined CHAOS+AMOS scenario.

Model	Params (M)	FiLM (%)	Size (MB)	Fwd. (ms/Patch)	Fwd. + Bwd. (ms/Patch)	Inf. (s/vol.)	Peak GPU Mem. (GB)
U-Net	5.60	0.00	21.37	50.00	126.79	0.812	1.30
U-Net + FiLM	5.66	1.05	21.59	50.22	127.77	0.810	1.37
nnU-Net	30.71	0.00	335.76	135.15	325.67	4.160	1.78
nnU-Net + FiLM	30.85	0.47	336.32	141.70	340.29	4.357	1.96

## Data Availability

The data presented in this study are available on request from the corresponding author.
